# Kinome Analysis of Cattle Peripheral Lymph Nodes to Elucidate Differential Response to *Salmonella* spp.

**DOI:** 10.3390/microorganisms10010120

**Published:** 2022-01-07

**Authors:** Ryan J. Arsenault, Tyson R. Brown, Thomas S. Edrington, David J. Nisbet

**Affiliations:** 1 Department of Animal and Food Sciences, University of Delaware, 044 Townsend Hall, Newark, DE 19716, USA; 2 Food and Feed Safety Research Unit, USDA-ARS, 2881 F & B Road, College Station, TX 77845, USA; Tyson_Brown@cargill.com (T.R.B.); tedrington@diamondv.com (T.S.E.); david_nisbet@yahoo.com (D.J.N.)

**Keywords:** *Salmonella*, dairy, beef, innate immunity, kinome

## Abstract

*Salmonella* spp., contained within the peripheral lymph nodes (PLNs) of cattle, represents a significant source of contamination of ground beef. Herein is the first report where species-specific kinome peptide arrays designed for bovine biology were used to further the understanding of *Salmonella* spp. within these PLNs. For the purpose of this research, multiple comparisons of sub-iliac lymph nodes were made to include nodes from feedlot cattle that were infected with *Salmonella* spp. to those that were non-infected; seasonal differences in feedlot cattle harvested in either August or January; cull dairy cows compared to feedlot cattle; and PLNs from cattle experimentally inoculated with *Salmonella* spp. versus naturally infected animals. The first comparison of *Salmonella*-positive and -negative PLNs found that considering the kinotypes for these animals, the major distinguishing difference was not the presence or absence of *Salmonella* spp. in the PLNs but the concentration. Further, the majority of pathways activated were directly related to immune responses including innate immunity, thus *Salmonella* spp. within the PLNs activates the immune system in that node. Results from the comparison of feedlot cattle and cull dairy cows suggests that a *Salmonella* spp.-negative animal, regardless of type, has a more consistent kinome profile than that of a *Salmonella* spp.-positive animal and that the differences between feedlot and cull dairy cattle are only pronounced when the PLNs are *Salmonella* spp. positive. PLNs collected in the winter showed a much more consistent kinome profile, regardless of *Salmonella* status, suggesting that in the winter these cattle are similar, and this is not affected by the presence of *Salmonella* spp., whereas significant variability among kinotypes was observed for PLNs collected in the summer. The most distinct clustering of kinotypes observed in this study was related to how the animal was infected with *Salmonella* spp. There were significant differences in the phosphorylation state of the immune response peptides between experimentally and naturally infected animals, suggesting that the immune system is activated in a significantly different manner when comparing these routes of infection. Increasing our understanding of *Salmonella* spp. within cattle, and specifically within the PLNs, will ultimately help design effective pre-harvest intervention strategies as well as appropriate experimentation to validate those technologies.

## 1. Introduction

*Salmonella* spp. are a common gastrointestinal resident in both dairy and feedlot cattle in the southwestern United States [1,2,3]. More recently in cattle, it was reported that the peripheral lymph nodes (PLNs) often harbor *Salmonella* spp., and unless removed by trimming, constitute a significant food safety threat [4,5,6,7]. These nodes are often located within adipose tissue, and therefore are impervious to in-plant anti-bacterial intervention strategies implemented at harvest. Recent research has reported the prevalence and concentrations of *Salmonella* spp. within these nodes in cattle at harvest in the United States and documented differences due to region, season and cattle type [5]. Utilizing a relatively large number of PLN samples collected from other projects representing cattle slaughtered in the southwestern United States, a significant amount of data was generated from each sample, and multiple, biologically relevant comparisons were conducted.

The method used to carry out these comparisons was the species-specific kinome peptide arrays designed for bovine biology. These arrays have been successfully used in a number of contexts, including the study of Johne’s Disease [8], prion-induced bovine spongiform encephalopathy or mad cow disease [9], and to examine stress responses [10]. The principle behind the technique involves designing a peptide array of kinase target sites, specifically designed to match the amino acid sequence in the species of interest. By exposing a cellular lysate to the array, the active kinases phosphorylate their respective kinase target sequences, and this phosphorylation is then visualized and the level of peptide phosphorylation measured. Data generated includes the phosphorylation events carried out by active kinases within a tissue sample. Since phosphorylation-mediated cellular signal transduction by kinase enzymes is the central mechanism for controlling most cellular functions and processes, the data provides a valuable window into animal biology.

In the study described herein, we have performed two firsts in the field of kinomics, the study of signaling within lymphoid tissue and the analysis of samples taken from cattle both naturally and experimentally infected with *Salmonella* spp. We have taken a global perspective on the kinome by considering a large kinase phenotype or “kinotype” of the individual animals, clustering them in different combinations, in an attempt to understand their relationship to one another. We have also categorized the individual peptide results that represent the kinotype into functional groupings, both phosphorylation-based signaling pathways and biological processes.

As a starting point, we compared sub-iliac lymph nodes from feedlot cattle that were infected with *Salmonella* spp. to those that were non-infected. As *Salmonella* spp. are more prevalent in the PLNs in the summer and early fall versus the winter [5], we compared the sub-iliac lymph nodes from feedlot cattle harvested in either August or January. Cull dairy cows have been reported to have a lower prevalence of *Salmonella* spp. in the PLNs compared to feedlot cattle [5]. Therefore, a third comparison was conducted with sub-iliac lymph nodes collected from feedlot cattle and cull dairy cows. In order to further investigate the routes of infection, persistence of *Salmonella* spp. within the PLNs, and evaluate potential intervention strategies, our laboratory developed an animal model for experimental infection [11,12]. While successful, some differences between experimentally and naturally infected cattle exist, and therefore the final comparison examined PLNs from cattle experimentally inoculated with *Salmonella* versus naturally infected animals. Relative differences or similarities from these comparisons may illuminate the most biologically relevant differences between these animals in terms of animal health and susceptibility to disease.

## 2. Materials and Methods

### 2.1. Lymph Node Tissue Collections

The sub-iliac lymph node was utilized in all comparisons and collected in commercial abattoirs, with the exception of the experimentally infected animals. Those cattle were Holstein steers, experimentally infected as described previously [12] and the sub-iliac lymph nodes removed at necropsy conducted at the United States Department of Agriculture—Agricultural Research Service (USDA-ARS) facilities with all procedures pre-approved by the USDA-ARS Animal Care and Use Committee (ACUC) committee, ACUC No. 2013001. For the first comparison, *Salmonella*-positive and -negative lymph nodes were obtained from feedlot cattle, 4 *Salmonella*-positive and 6 *Salmonella*-negative, one node per animal. Cattle originated from a single, commercial feedlot, were housed within the same pen and harvested the same day. *Salmonella*-positive nodes all had low concentrations, averaging 0.3 colony forming units (CFU) (log_10_)/g lymph node, while *Salmonella*-negative nodes were negative following both quantitative and qualitative culture. The second comparison examined differences between cattle originating from a commercial feedlot and a commercial dairy (cull dairy cows). Lymph nodes from six cull Holstein cows were utilized, three of which were *Salmonella* positive (qualitative culture only). Six of the fed cattle lymph nodes from the first comparison were utilized again for the second comparison, three of which were *Salmonella* positive [average concentration 0.2 CFU (log_10_)/g lymph node] and three nodes that were negative. The third comparison examined seasonal differences in lymph nodes collected from feedlot cattle in the summer (August) or winter (January). Cattle originated from the same commercial feedlot. Five lymph nodes represented the summer collection, all *Salmonella* positive quantitatively and qualitatively, with an average concentration of 1.4 CFU (log_10_)/g lymph node, while 12 nodes were used for the winter collection, six of which were *Salmonella* positive [average concentration 0.3 CFU (log_10_)/g lymph node]. As most all of the lymph nodes were *Salmonella* positive in the summer, these animals were categorized as having a higher [1.5–2.8 CFU (log_10_)/g lymph node] or lower [0.1–0.2 CFU (log_10_)/g lymph node] concentration of *Salmonella,* whereas winter animals were classified as being either *Salmonella* positive or negative. The final comparison examined lymph nodes from cattle experimentally infected with *Salmonella* to those that acquired the *Salmonella* naturally. Six sub-iliac lymph nodes, all *Salmonella* positive [average concentration of 1.2 CFU (log_10_)/g lymph node], were obtained from experimentally infected Holstein steers and compared to 6 *Salmonella*-negative nodes collected during the winter and used in the seasonal comparison above.

### 2.2. Peptide Arrays

A small portion (approx. 2 g) of each node was removed and frozen at −80 °C for later analysis within 24 h of slaughter. Lymph nodes were processed for concentration and prevalence of *Salmonella* as described previously [13]. Tissue samples were weighed to obtain a consistent sample size for the array protocol. Samples were homogenized by a bead based Bead Bug Microtube Homogenizer (Benchmark Scientific, Edison, NJ, USA). The tissue sample and buffer were placed in a microtube containing lysis beads and 100 µL of lysis buffer [20 mM Tris–HCl pH 7.5, 150 mM NaCl,1 mM EDTA, 1 mM Ethylene glycol tetraacetic acid (EGTA), 1% Triton X-100, 2.5 mM sodium pyrophosphate, 1 mM Na_3_VO_4_, 1 mM NaF, 1 μg/mL leupeptin, 1 g/mL aprotinin and 1 mM phenylmethylsulphonyl fluoride (all products from Sigma Aldrich, St. Louis, MO, USA), unless indicated]. The tube was placed in the homogenizer at maximum speed for 30 s. Following homogenization, the peptide array protocol was carried out as reported previously [14] with alterations described by Arsenault and colleagues [15].

### 2.3. Data Analysis for Peptide Array

Data normalization, statistics and clustering analysis were performed for the peptide arrays as described by Li and co-workers [16] using the Platform for Intelligent, Integrated Kinome Analysis version 2 (PIIKA2) online software platform [17]. Gene Ontology (GO) and Kyoto Encyclopedia of Genes and Genomes (KEGG) pathway analysis were performed by uploading the statistically significant peptide lists to the Search Tool for the Retrieval of Interacting Genes (STRING) [18].

## 3. Results

A heatmap and cluster diagram was generated utilizing all of the lymph tissue samples (across all comparisons) and is presented as Supplementary Figure S1. When combining all of the results, it is difficult to provide biological context to such a large and diverse data set in one analysis. Thus, we have broken down this data into separate comparisons that are described in detail below.

### 3.1. Comparison I—Salmonella-Positive vs. -Negative Sub-Iliac Lymph Nodes Obtained from Feedlot Cattle

**Figure 1:**
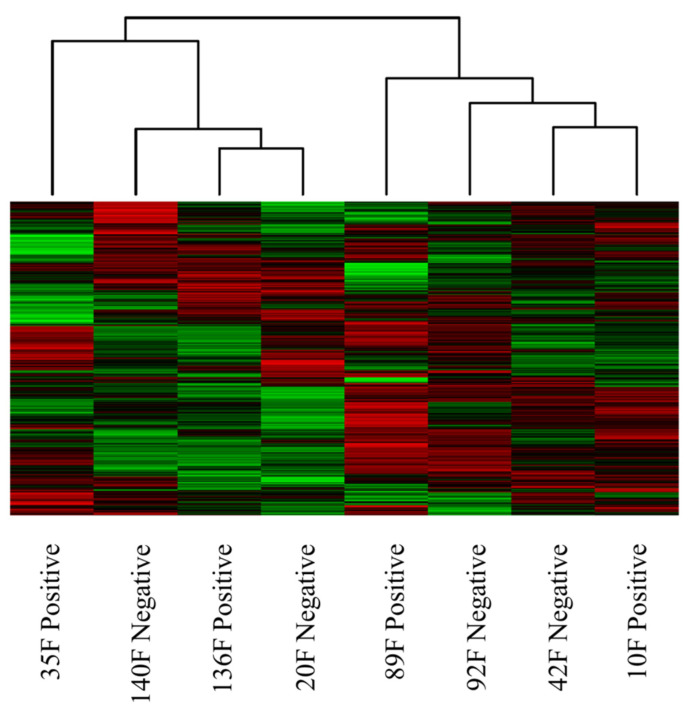
Heatmap and clustering analysis of feedlot cattle that were culture positive or negative for *Salmonella* in the sub-iliac lymph node, samples clustered based on the relative similarity of the kinome profiles. The interspersing of *Salmonella*-positive and -negative animals indicates that infection status is not the overriding differentiator of the two groupings. Each column represents a tissue sample; each row is a peptide. Red represents relative increase in phosphorylation and green relative decrease.

Sub-iliac lymph nodes were collected from feedlot cattle and *Salmonella* infection status was determined (positive or negative) (Table 1). A set of positive and negative tissue samples were analyzed by kinome peptide arrays. The heatmap shows the relative increase (red) or decrease (green) in phosphorylation of the individual peptides on the array following exposure to the PLN lysate. The clustering (lines at the top of Figure 1) shows the relative similarity or difference between the entire kinome profile of the individual animals; the shorter the height of the lines connecting two animals, the greater the similarity between them. The heatmap and clustering analysis (Figure 1) shows two distinct clusters, with two outliers in each cluster. Both outliers (35 F and 89 F) were animals with *Salmonella*-positive lymph nodes. The remaining animals were more similar to each other (thus the shorter linkage lines), and within these tighter clusters there were two negative animals and one positive animal.

When considering the kinotypes above (Figure 1), we are looking at differences in the kinome profiles that are not solely due to the *Salmonella* infection status, these kinome profiles also include normal basal level kinase activities. In order to focus our subsequent analysis on changes in kinome profiles due to the *Salmonella* infection, we combined the *Salmonella*-positive animals and the *Salmonella*-negative animals to generate representative kinome profiles of each. Comparing these profiles generated a fold-change (fold change = *Salmonella*-positive signal/*Salmonella*-negative signal) and *p*-value (significance of difference between positive and negative signal) for the individual peptides that make up the kinome profile. The resultant data should be primarily measures of kinome changes attributable to the *Salmonella* in the lymph nodes. The signal from both the basal kinase activities and the animal-to-animal variation should be reduced. Using only these statistically significant peptides (*p* < 0.05), we further analyzed this data for patterns. Table 2 shows the top 12 Kyoto Encyclopedia of Genes and Genomes (KEGG) pathways generated by the STRING online data analysis tool. Intracellular signaling pathways are key to understanding animal biology, can be induced by any number of stimuli, and affect nearly all cell and tissue processes (KEGG is a database of these pathways).

A GO Biological Process analysis using the STRING data analysis tool was also performed. These GO terms are more broad-based categories of biological function than the KEGG pathways but still involve the protein intermediates that carry out signaling within a cell. The results in Table 3 show the statistically significant differential phosphorylation signaling involved in immune response changes between cattle with *Salmonella*-positive and -negative lymph nodes. The top four terms are directly related to immune responses including innate immunity, thus *Salmonella* within the PLNs—in this case, the sub-iliac lymph node—activates the immune system in that node to a significant degree.

### 3.2. Comparison II—Sub-Iliac Lymph Nodes Collected from Feedlot or Cull Dairy Cattle at Slaughter

**Figure 2:**
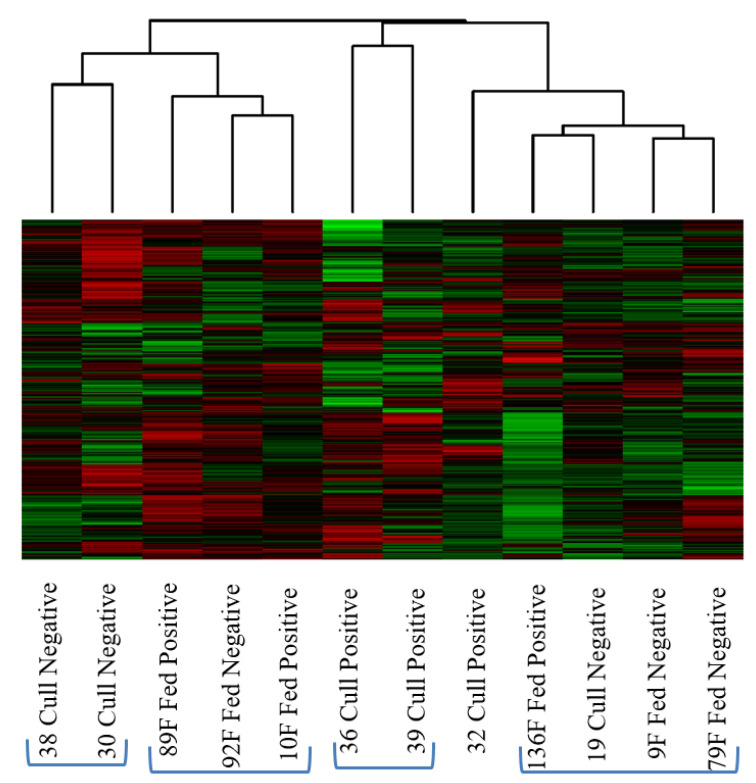
Heatmap and cluster analysis of sub-iliac lymph nodes collected from feedlot (fed) and cull dairy cattle (cull) at slaughter that are either *Salmonella* positive or negative. Clusters are based on their relative similarity. Each column represents a tissue sample; each row is a peptide. Red represents relative increase in phosphorylation and green relative decrease.

Within each class of cattle (feedlot or cull dairy), sub-iliac lymph nodes were collected that were *Salmonella* positive and negative (Table 4). The heatmap and cluster analysis (Figure 2) illustrates what appears to be an interspersing of beef and dairy cattle as well as *Salmonella*-negative and -positive individuals. However, on closer inspection, there is a separation between the feedlot and cull dairy cattle. Moving from left to right in Figure 2, there are two *Salmonella*-negative cull dairy (#38, #30), a cluster (2 of which are *Salmonella*-positive) of three feedlot animals (#89 F, #92 F, #10 F), two *Salmonella*-positive cull dairy (#36, #39), an individual cull dairy (#32, also *Salmonella* positive) and then a cluster of feedlot cattle (#136 F, #9 F, #79 F; two negative and one positive for *Salmonella*) containing one cull dairy *Salmonella*-negative individual outlier (#19). Cow #19 was from a different farm than the other cull cattle, which may be why it clusters differently. 

**Figure 3:**
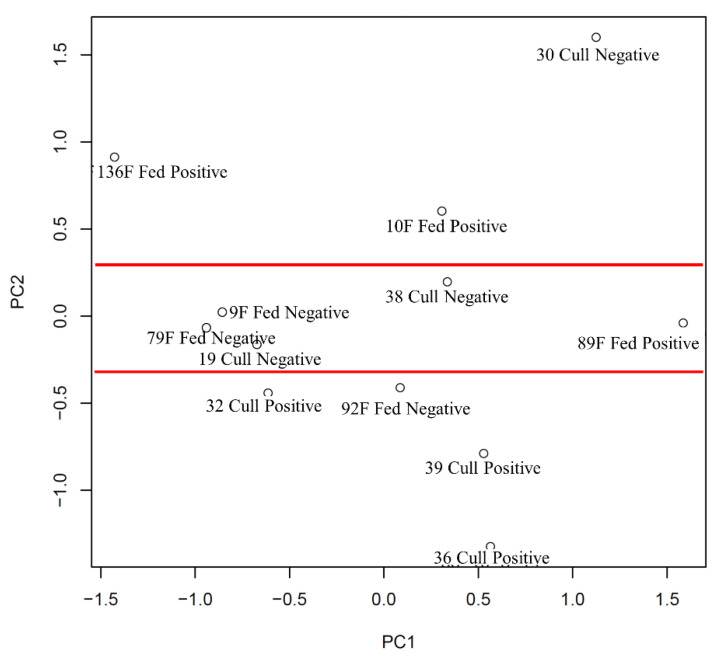
Principal component analysis (PCA) plot generated from sub-iliac lymph nodes collected from feedlot and cull dairy cattle at slaughter that were either *Salmonella* positive or negative. The kinome profiles of these animals were plotted by PCA. Between the red lines are all but one of the negative cows. SD% of PC1 = 18.21%; SD of PC2 = 15.47%.

Using a different method of data analysis, principal component analysis (PCA) to examine the above data, a clearer cluster pattern was generated. The PCA (Figure 3) shows that clustered around the center of the plot on the PC2 axis, there is a group of *Salmonella*-negative cattle (between the red lines), both feedlot and cull dairy animals. Only one individual animal that is *Salmonella* negative lies outside of this center grouping (cull dairy cow #30). Of interest is that in this PCA, the *Salmonella*-negative animals displayed a similar pattern while those that were *Salmonella* positive were broadly scattered across the plot. Table 5 shows the KEGG pathways and Table 6 shows the GO term biological processes, calculated by comparing feedlot/cull dairy cattle.

### 3.3. Comparison III—Season (Summer vs. Winter)

**Figure 4:**
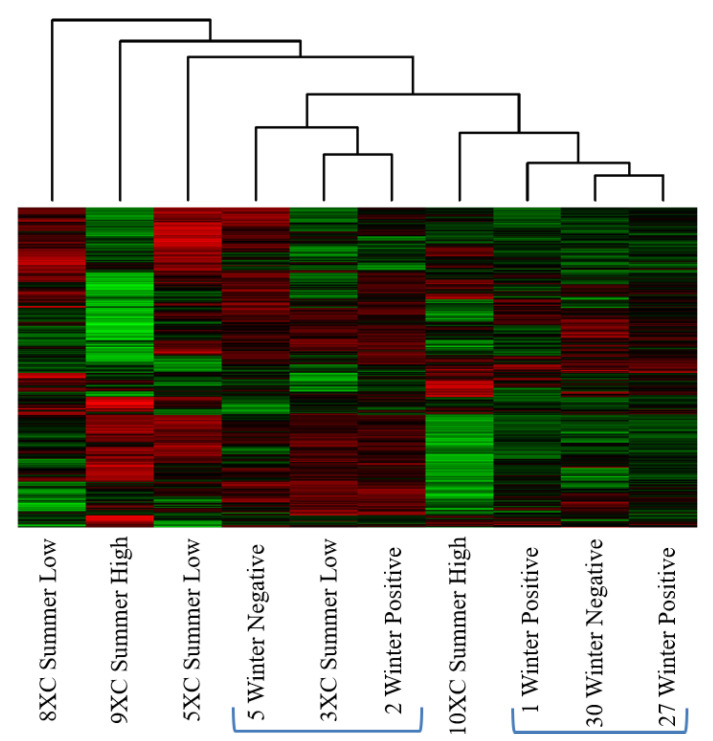
Heatmap and clustering analysis of summer cattle that had either high or low concentrations of *Salmonella* in the sub-iliac lymph nodes, and winter cattle, that were positive or negative for *Salmonella* in the sub-iliac lymph node. Clusters are based on the relative similarity of the kinome profiles. The two clusters of winter cattle and the interspersed and outlier summer cattle indicate a varied kinome in the summer animals. Each column represents a tissue sample; each row is a peptide. Red represents relative increase in phosphorylation and green relative decrease.

In this comparison, we examined sub-iliac lymph node tissue collected from animals slaughtered in the summer and winter months and also factored in the actual concentration of *Salmonella* within the PLNs (Table 7). The heatmap and cluster analysis of the kinome data (Figure 4) shows that the lymph tissue collected from the summer animals was highly variable in their respective kinotypes. On the left side of Figure 4, three of the five summer individuals are outliers and do not cluster with any other individuals. Sample 10XC Summer High also sits outside of the two clusters generated by the winter animals. The winter animals are more similar to each other and they form two clusters, one of which contains the summer animal 3XC Summer Low.

**Figure 5:**
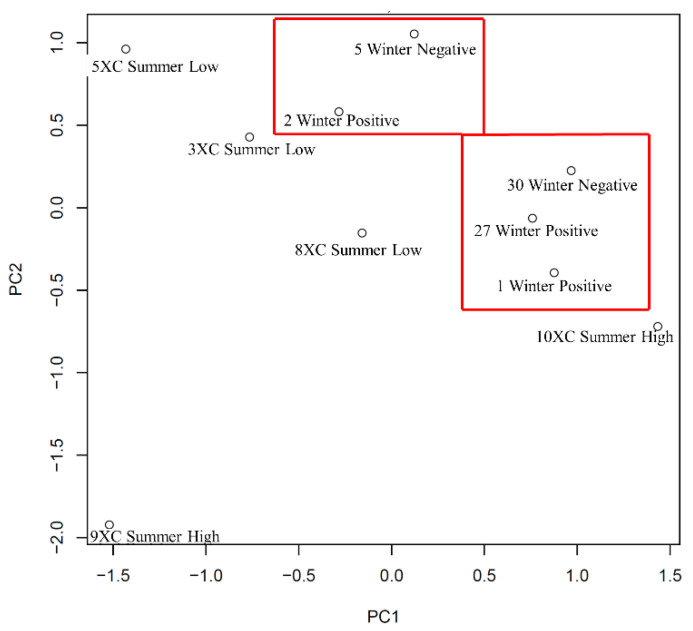
Principal component analysis (PCA) plot generated from the sub-iliac lymph nodes collected from cattle at slaughter in the summer (high or low *Salmonella* concentrations) and winter (*Salmonella* positive or negative). The kinome profiles of these animals were plotted by PCA. SD% of PC1 = 22.42%; SD of PC2 = 19.34%.

The PCA plot (Figure 5) produces results similar to those observed in the heatmap and cluster analysis above. The animals, #5 Winter Negative and #2 Winter Positive are closely grouped as are the other three winter animals [similar based on the PC1 axis and quite similar based on PC2 axis (see red boxes)]. The summer individuals are all very distinct on both axes, further confirming the variable kinotypes between these lymph nodes collected in the summer.

As observed in the feedlot versus cull dairy cattle comparison, both the KEGG pathway analysis (Table 8) and the GO Biological Process analysis (Table 9) generated results with fewer immune response elements than the first comparison of *Salmonella*-positive and -negative samples.

### 3.4. Comparison IV—Experimental vs. Natural Infection

**Figure 6:**
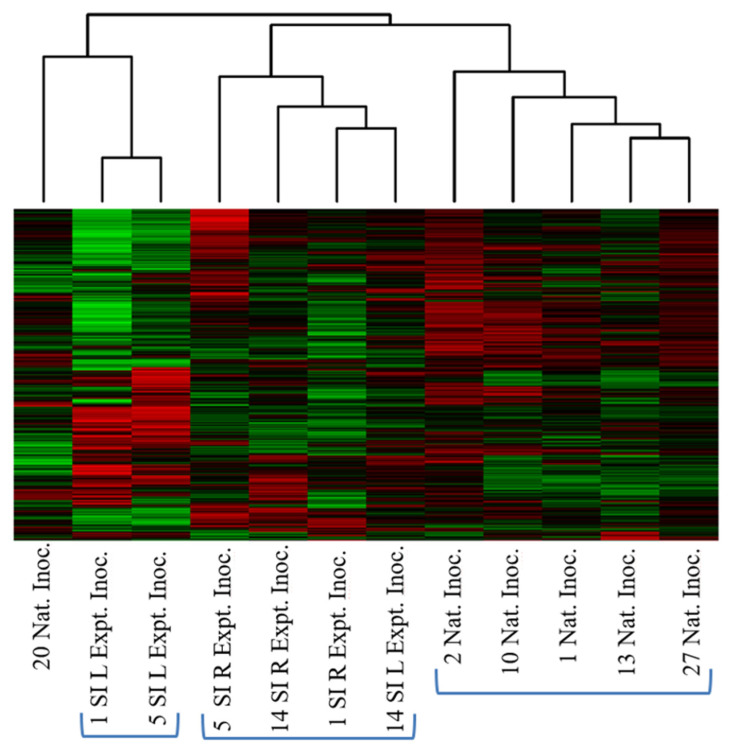
Heatmap and clustering analysis of peripheral lymph nodes from cattle that were experimentally or naturally infected with *Salmonella* and clustered based on the relative similarity of the kinome profiles. Two obvious clusters of natural and experimental infection are observed to the right and center of the figure. Each column represents a tissue sample; each row is a peptide. Red represents relative increase in phosphorylation and green relative decrease.

In this comparison, we generated and analyzed the kinome data from PLNs of cattle that were experimentally infected with *Salmonella* to cattle with PLNs that were naturally infected (Table 10). From the heatmap and clustering of this data (Figure 6), three distinct groupings of individuals were observed. The cluster on the right is composed of naturally infected animals; the cluster in the center represents experimentally infected animals; and the final cluster on the left are two similar, yet outlying, experimentally infected animals; followed on the far left by a single naturally infected animal. 

**Figure 7:**
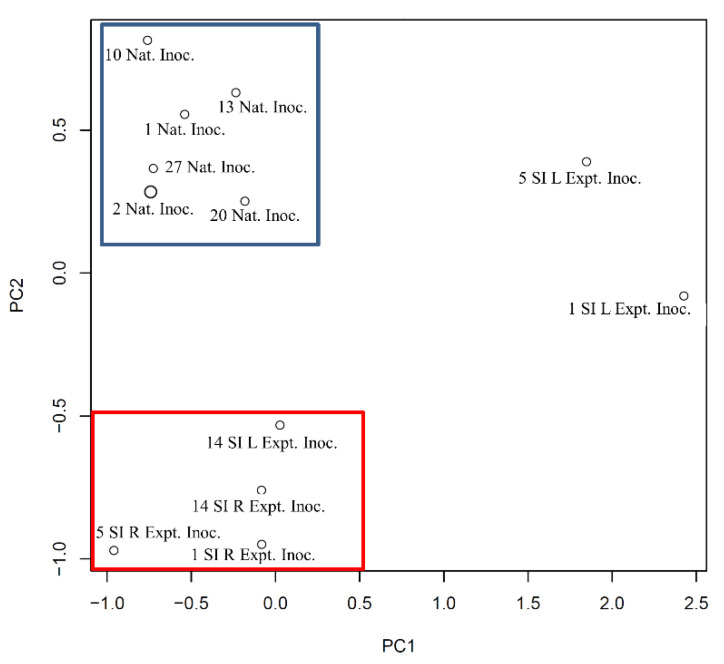
Principal component analysis (PCA) plot from sub-iliac lymph nodes collected from experimentally and naturally infected cattle at slaughter. The kinome profiles of these animals were plotted by PCA. SD% of PC1 = 24.35; SD of PC2 = 14.77%.

The PCA plot (Figure 7) was broadly similar to the heatmap and cluster analysis. The cluster of naturally infected animals was very clear (blue box), as was the cluster of experimentally infected animals (red box); aside for the two outliers identified on the heatmap, (5FEML Expt. Inoc. and 1FEML Expt. Inoc.). Interestingly, the 20 Nat. Inoc. animal was not an outlier in the PCA as it was in the heatmap above, and this individual associates closely with the other naturally infected animals.

The differences between the KEGG pathway analysis (Table 11) and the GO Biological Process analysis (Table 12) are of note in this study. In the KEGG analysis, the top three pathways were not immune function related, contrary to what was observed in the GO analysis where the top three pathways were related to immune function and when combined represent 652 peptides on the array.

## 4. Discussion

To our knowledge, this is the first research reporting the use of kinome peptide arrays to elucidate the differences in response to *Salmonella* acquisition by the PLNs of cattle. Our first comparison looked for differences in sub-iliac nodes from feedlot cattle that were either *Salmonella* positive or negative. Based on the heatmap and clustering of this data, we observed that the *Salmonella* infection state (positive or negative) of the lymph node was not the over-riding factor driving how the individual kinotypes cluster (Figure 1). Within the two predominant clusters, both negative and positive animals were represented. However, the two positive animals (10 F and 136 F) that clustered with the negative animals had the lowest concentrations of *Salmonella* (Table 1). It is common in the “omics” analysis of outbred individual animals that differences in individuals are greater than the response to a given treatment or condition. In addition, we have found in previous studies that individual-to-individual differences are greater at the protein level than at the transcript level [19]. This is akin to how differences at the transcript level are significantly greater between individuals or species than differences at the genome level. It may be that a higher concentration of *Salmonella*, such as that observed in animals 35 F and 89 F, is required to alter the clustering of the kinotype.

By combining data (to create two representative kinome profiles of *Salmonella*-positive and -negative animals) and comparing the two infection states, any observed changes in peptide phosphorylation as a likely result of *Salmonella* would be apparent, and changes due to other factors were eliminated from the analysis due to their inherent variability between individuals. The positive lymph nodes (from animals 136 F and 10 F) both contained lower concentrations of *Salmonella* (Table 1), which is likely why they clustered with the negatives. This data indicates that considering the kinotypes for these animals, the major distinguishing difference was not the presence or absence of *Salmonella* in the PLNs but the concentration. This new profile, containing phosphorylation fold changes and their associated *p*-values, was then input into an online database (STRING) to organize and categorize the data. The KEGG pathway analysis demonstrated that the general MAPK signaling pathway was the most significantly altered pathway (Table 2). Many of the pathways in Table 2 are involved in immune response, not surprising given the differential bacterial infection state of the comparison. The MAPK signaling pathway is involved in many cellular functions, including immunity, and is a large pathway, containing many protein intermediates that transmit signals within the cell, so it ranks highest on the list. Pathways in cancer is a very broad description involving many MAPKs, growth and cell differentiation signaling intermediates, and that is why it is highly significant in the analysis; however, it does not represent any cancer-causing potential of *Salmonella*. The presence of a cancer pathway does not mean that *Salmonella* infection and cancer are similar, only that they both cause a wide variety of changes in similar types of protein signaling molecules within a cell. There were also a number of immune-specific pathways that were altered due to *Salmonella* presence within the PLNs, including T cell receptor and Toll-like receptor signaling pathways, supporting that both innate and adaptive immune responses were engaged by the *Salmonella*. The GO Biological Process analysis showed a more significant immune response when comparing *Salmonella*-positive to -negative animals than the KEGG analysis, as the top 4 most significant biological processes were all related to immune function (Table 3). As might be expected, there was a difference in the immune response of *Salmonella*-infected when compared to non-infected animals. The innate immune response was the most significant process affected in these results and *Salmonella* is a well-known inducer of the innate inflammatory response in cattle [20,21,22]. This result would be expected as *Salmonella* infection is dependent on a host inflammatory response for successful invasion [21] and our results demonstrate that *Salmonella* within the PLNs engages in a localized immune response.

In the second comparison, we examined *Salmonella*-positive and -negative sub-iliac lymph nodes collected from feedlot steers and cull dairy cattle at slaughter. Previously, it was reported that cull cows (including cull dairy) were less likely to harbor *Salmonella* in the PLNs than cattle originating from feedlots [5]. The heatmap and clustering analysis of the data did not provide clear groupings, but did indicate some interesting trends (Figure 2). There appeared to be some clustering based on animal type (feedlot or cull dairy); however, some individual members of each were found over all portions of the cluster map. If cattle type is ignored and the data reorganized by *Salmonella* status, the same heatmap cluster figure does not show a clear clustering pattern, but does indicate more similarity between individual animals within the same *Salmonella* status than when cattle type was compared. As we observed in Figure 1 there is some clustering of low dose *Salmonella*-positive animals with the *Salmonella*-negative animals (cow 136 F) (Figure 2). The PCA plot resulting from the kinome data from these individual animals provides a better picture of how the kinotypes are related to one another (Figure 3). The *Salmonella*-negative animals clustered in the center of the plot (between red lines) and there was more variation among these negative animals on the PC1, as compared to the PC2 axis. There was a single negative outlier, while the positive animals showed substantial separation on both PC1 and PC2 axes. There was also substantial separation among feedlot and cull dairy animals that were *Salmonella* positive on both PC1 and PC2 axes. Thus, we conclude that the overriding similarity was a result of *Salmonella* status (negative animals) and not cattle type. This suggests that a *Salmonella*-negative animal, regardless of type, has a more consistent kinome profile than that of a *Salmonella*-positive animal. The differences between feedlot and cull dairy cattle only become pronounced when the animals are *Salmonella* positive, perhaps due to their differential immune response to the infection. Not surprising, as personal communications with commercial beef packers after this research was conducted suggests that cull dairy cattle lymph nodes often harbor *Salmonella*, in contrast to early research [5].

The data indicates that the response to invading *Salmonella* is variable and thus the susceptibility to *Salmonella* infection may not manifest itself until after PLN exposure to *Salmonella*. In other words, it is difficult to predict how a dairy or beef animal will respond to *Salmonella* prior to exposure. This difference in susceptibility only appeared following a *Salmonella* infection, as indicated by a separation on the PCA plot (PC2 axis: positive fed beef cattle above 0.0 and positive cull dairy at or below −0.5). In this study, the two types of cattle are very different in terms of age, breeding, farm of origin, management, and other factors, which might explain this different response to *Salmonella* infection. This differential response to *Salmonella* only appeared following an infection of the PLNs and is likely due to a substantial difference in immune response, possibly due to the large differences in metabolic activity affecting immunity. Dairy and beef cattle are bred to emphasize different metabolic traits, and this can affect immunity. Evidence for this can be seen in the KEGG pathway (Table 5) and GO Biological Process (Table 6) results generated from the kinome data. The KEGG pathway analysis showed that the majority of the immune response pathways ranked lower on the list of top pathways, and that a smaller number of peptides were involved in the immune pathways than was observed in the *Salmonella*-infected versus non-infected comparison. This is not an unexpected result, as in the first comparison, we compared *Salmonella*-negative and -positive animals to determine the effects of the infection, and in this comparison, we are looking for differences between feedlot and cull dairy cattle. In addition, the number of protein metabolism peptides changed between the cull dairy cows and feedlot cattle and is greater than that observed in the comparison of *Salmonella* status. As beef cattle and dairy cattle have been bred for very different purposes, centered on either muscle growth or milk production, a difference in protein metabolism ultimately resulting in a different immune response is a logical result. The differences between the two types of cattle are not necessarily solely due to immune response to *Salmonella*. There are several peptides, pathways and processes involved in immune function listed in Table 5 that are distinct for the two types of cattle. These differences could have presented following exposure to *Salmonella,* or may have been present at all times, thus explaining the difference in susceptibility of these two types of cattle to *Salmonella* infection of the PLNs. Our results indicate that further analysis should focus on how the uninfected cull dairy and beef cattle differ which may point to pre-infection mechanistic reasons, perhaps related to age of first exposure in different production environments, for their different susceptibility and response to *Salmonella*. This would allow predictions of what animals are likely to clear *Salmonella* or not.

In the third comparison, the heatmap and cluster analysis results (Figure 4) from sub-iliac lymph nodes collected from feedlot cattle at slaughter in the summer and winter months were similar to the feedlot and cull dairy comparison results, in that the clustering appeared disparate while the PCA plot provided more insight into the relative similarities among the individuals (Figure 5). Three winter samples clustered together, while the remaining individuals were randomly clustered on the plot, with three summer individual outliers. Taking the PCA plot into consideration, two relative groupings of winter individuals emerged (and some summer cattle), regardless of *Salmonella* infection status or concentration (Figure 5). These results indicate that the winter samples share a relatively common kinotype, while the samples collected in the summer were more variable. The question may arise as to the value of combining the summer results in order to generate a representative summer kinotype. Recall that the nature of the analysis takes into account individual variability and assigns a high *p*-value to those peptides with a greater variability among the individuals of a group. Thus, the peptides that come out of the analysis as significant are those that are consistent among individuals and significantly different from the winter values. A more in-depth analysis of the individual peptides and their changes in phosphorylation will be required to further elucidate the mechanism behind the differences among the summer and winter lymph nodes. It is possible, as we are considering both immune and metabolic responses, that the winter cattle are more similar because they are engaging similar metabolic processes in the cooler winter months which may have the effect of generating similar kinotypes.

The final comparison examined PLNs from cattle that were experimentally inoculated with *Salmonella* to those that were naturally infected and illustrates a more clearly defined clustering than any of the previous comparisons. The heatmap and cluster analysis showed that all but one of the naturally infected animals clustered together, while the experimentally infected cattle clustered into two distinct groups (Figure 6). The PCA plot again showed the clearest relationship between the individual animal kinotypes (Figure 7). The same outliers were observed in the experimentally inoculated animals, while no outliers were observed among the PLNs from the naturally infected cattle. The above results are of interest to those working with experimental- infection animal models and needs to be considered in the context of experimental design and data interpretation when it comes to infectious disease research. Such striking differences, apparently due to the method of infection and possibly the dose of infectious agent, may have implications on the subsequent results of animal experiments and this data may contribute to an increased understanding of the differences between animal models using experimental infection versus those that incorporate naturally infected animals. The results presented herein demonstrate that there were significant differences in how the cattle responded to the different modes of infection. The KEGG pathways and biological processes that were generated from the comparison of the two groups of cattle were largely similar to the previously discussed results, and a more detailed analysis of the individual peptides is required to better understand the mechanistic differences between them. Of note is that the T cell receptor signaling (KEGG pathway, Table 11) and the Fc receptor signaling pathway (biological process, Table 12) are both significantly different between the two groups. This was similar to the results observed when we compared the *Salmonella* infected to the non-infected lymph nodes and may suggest potentially large pathway differences in the adaptive immune response between experimentally and naturally infected cattle whereas there were large, broad differences in the innate immune response. These differences may be of a similar magnitude to the changes observed among *Salmonella*-positive and -negative cattle. This suggests that the adaptive immune responses are different whereas the same innate immune receptors are engaged in both natural and experimentally infected animals, but the downstream effects may be greater in one than the other. It is important to note that these differences appear when comparing PLNs that are all infected with *Salmonella*, only the mode of infection is different. It is important to consider, however, that there were differences other than route of infection. The animals used in the experimental-infection experiments were Holstein steers, younger than the naturally infected, feedlot steers composed of beef breeds, and housed, fed and managed differently. Thus, the separation of individuals observed may include other factors in addition to the mode of infection. Additionally, it is not possible to determine whether the PLNs of the experimentally infected animals had been infected naturally prior to experimental infection. That said, based on difficulties encountered in our laboratory in replicating the *Salmonella* concentrations observed in naturally infected cattle at slaughter using experimental inoculation, and the anecdotal observations of the ease at which cattle naturally acquire *Salmonella* in the PLNs, differences very likely exist. A comparison of the hundreds of phosphorylation events that are different among the two pairs of cattle is in order to fully characterize how *Salmonella*-infected vs. non-infected lymph nodes relate to experimental versus natural infection.

## 5. Conclusions

The various analyses and group comparisons conducted herein present a broad overview of the power of kinome analysis, animal kinotyping and peptide phosphorylation comparative analysis. In comparing PLNs that are *Salmonella* positive to those that are culture negative, it was demonstrated that individual animals display different kinome profiles centered on innate and adaptive (T cell receptor) immune responses to *Salmonella*. These cellular signal transduction changes brought about due to bacterial infection of the lymph nodes has never been considered at an active protein level. In comparing feedlot to cull dairy cattle, we illustrated that the *Salmonella* infection state was more important than cattle breed or farm of origin. Additionally, it was the *Salmonella* infection and subsequent response that separates these two cattle types, as in their non-infected (*Salmonella*-negative) state they looked remarkably similar at the kinome level. Understanding this separation following infection may help us understand why these two cattle types may have a differential susceptibility to *Salmonella* acquisition by the PLNs. In contrast to the feedlot and dairy cattle comparison, the summer/winter lymph node comparison appeared to be defined by season more than infection status. Animals sampled in the winter, regardless of *Salmonella* status, were more alike than cattle sampled in the summer, which were widely variable in their response. This summer variation also appeared independent of *Salmonella* concentration within the lymph node. Using these same PLNs, a representative summer and winter cattle kinotype was generated to show why different seasons appear to affect susceptibility to disease, with results suggesting that a significant immune component is involved. Finally, lymph nodes from experimentally inoculated cattle were distinct in their kinotype as compared to naturally infected animals. Further research into these differences is warranted. These results could have significant implications for animal disease experimentation as well as pre-harvest food safety research, as much of this research is conducted on experimentally infected animals and then applied to naturally infected animals. Further analysis of this data should increase our understanding of these differences, so that future research utilizing experimentally infected animals will be properly analyzed and interpreted.

## Figures and Tables

**Table 1:** Details of cattle positive or negative for *Salmonella* in the sub-iliac lymph node collected for kinome analysis.

				*Salmonella*	
Sample ID	Date	Source/ Cattle Type	Season	Concentration (CFU (log_10_)/g Lymph Node)	Prevalence
20 F	8/27/2014	Feedlot/ Holstein	summer	neg	neg
136 F	8/27/2014	Feedlot/ Native	summer	0.1	pos
140 F	8/27/2014	Feedlot/ Native	summer	neg	neg
79 F	8/27/2014	Feedlot/ Native	summer	neg	neg
42 F	8/27/2014	Feedlot/ Holstein	summer	neg	neg
89 F	8/27/2014	Feedlot/ Native	summer	0.5	pos
10 F	8/27/2014	Feedlot/ Holstein	summer	0.1	pos
35 F	8/27/2014	Feedlot/ Holstein	summer	0.6	pos
92 F	8/27/2014	Feedlot/ Native	summer	neg	neg

**Table 2:** The top 12 KEGG pathways in the sub-iliac lymph nodes collected from feedlot cattle at slaughter that were culture positive or negative for *Salmonella*. The list of represented pathways was generated by taking the statistically significant peptides and inputting this peptide list into the online STRING database. Presented here are the top 12 pathways from the input data. The bold pathways are those involved in the immune response (total immune peptides: 184).

KEGG ID	Pathway Name	# Peptides	*p*-Value (FDR)
hsa04010	MAPK signaling pathway	48	4.4.49 × 10^−36^
hsa05200	Pathways in cancer	51	8.99 × 10^−36^
hsa04722	Neurotrophin signaling pathway	34	3.18 × 10^−32^
**hsa04660**	**T cell receptor signaling pathway**	**31**	**2.71 × 10^−30^**
hsa04012	ErbB signaling pathway	28	2.99 × 10^−29^
**hsa04510**	**Focal adhesion**	**37**	**1.00 × 10^−28^**
hsa04910	Insulin signaling pathway	31	1.83 × 10^−27^
**hsa04620**	**Toll-like receptor signaling pathway**	**28**	**5.35 × 10^−27^**
hsa05215	Prostate cancer	26	8.54 × 10^−26^
**hsa05162**	**Measles**	**28**	**1.75 × 10^−23^**
**hsa05152**	**Tuberculosis**	**30**	**2.75 × 10^−22^**
**hsa04062**	**Chemokine signaling pathway**	**30**	**1.18 × 10^−21^**

**Table 3:** The top 12 GO biological processes in sub-iliac lymph nodes collected from cattle at slaughter that were culture positive or negative for *Salmonella*. A list of representative GO terms was produced using the statistically significant peptides generated from the comparison of *Salmonella*-positive and -negative lymph nodes that were inputted into the online STRING database. The top 12 terms from the input data are listed. The pathways in bold are those involved in the immune response (total immune peptides: 544).

GO ID	Term	# Peptides	*p*-Value (FDR)
**GO:0045087**	**innate immune response**	**97**	**3.56 × 10^−56^**
**GO:0002764**	**immune response-regulating signaling pathway**	**72**	**1.04 × 10^−49^**
**GO:0050776**	**regulation of immune response**	**84**	**1.06 × 10^−47^**
**GO:0006955**	**immune response**	**101**	**3.69 × 10^−46^**
GO:0051246	regulation of protein metabolic process	116	7.34 × 10^−45^
GO:0009967	positive regulation of signal transduction	88	1.02 × 10^−42^
GO:0032268	regulation of cellular protein metabolic process	103	1.09 × 10^−42^
**GO:0048584**	**positive regulation of response to stimulus**	**101**	**1.18 × 10^−40^**
**GO:0071310**	cellular response to organic substance	103	1.61 × 10^−40^
GO:0023056	positive regulation of signaling	87	2.16 × 10^−40^
**GO:0002682**	**regulation of immune system process**	**89**	**5.98 × 10^−40^**
GO:0042325	regulation of phosphorylation	87	5.99 × 10^−40^

**Table 4:** Details of cattle cull or dairy, positive or negative for *Salmonella* in the sub-iliac lymph node, collected for kinome analysis.

				*Salmonella*		
Sample ID	Date	Source/ Cattle Type	Season	Concentration (CFU (log_10_)/g Lymph Node)	Prevalence	Other Info
30	8 October 2014	Cull/ dairy cows	Fall	neg	neg	Texas Farm 1
32	8 October 2014	Cull/ dairy cows	Fall	neg	pos	Texas Farm 1
38	8 October 2014	Cull/ dairy cows	Fall	neg	neg	Texas Farm 1
36	8 October 2014	Cull/ dairy cows	Fall	neg	pos	New Mexico
39	8 October 2014	Cull/ dairy cows	Fall	neg	pos	Texas Farm 1
19	8 October 2014	Cull/ dairy cows	Fall	neg	neg	Texas Farm 2
136 F	27 August 2014	Feedlot/ Native	summer	0.1	pos	Feedlot 1
79 F	27 August 2014	Feedlot/ Native	summer	neg	neg	Feedlot 1
89 F	27 August 2014	Feedlot/ Native	summer	0.5	pos	Feedlot 1
10 F	27 August 2014	Feedlot/ Holstein	summer	0.1	pos	Feedlot 1
92 F	27 August 2014	Feedlot/ Native	summer	neg	neg	Feedlot 1
9 F	27 August 2014	Feedlot/ Holstein	summer	neg	neg	Feedlot 1

**Table 5:** The top 12 KEGG pathways in the sub-iliac lymph nodes collected from feedlot and cull dairy cattle. A list of representative pathways was generated utilizing the statistically significant peptides from the comparison and inputting this peptide list into the online STRING database. Presented below are the top 12 pathways. Pathways in bold are involved in the immune response (total immune peptides: 141).

KEGG ID	Pathway Name	# Peptides	*p*-Value (FDR)
hsa04722	Neurotrophin signaling pathway	43	1.17 × 10^−43^
hsa05200	Pathways in cancer	58	1.25 × 10^−41^
hsa04910	Insulin signaling pathway	42	2.21 × 10^−41^
hsa04012	ErbB signaling pathway	35	3.80 × 10^−39^
hsa04010	MAPK signaling pathway	51	3.15 × 10^−38^
**hsa04660**	**T cell receptor signaling pathway**	**36**	**1.52 × 10^−36^**
**hsa04510**	**Focal adhesion**	**41**	**4.71 × 10^−32^**
**hsa04662**	**B cell receptor signaling pathway**	**27**	**1.25 × 10^−28^**
**hsa04062**	**Chemokine signaling pathway**	**37**	**1.75 × 10^−28^**
hsa05214	Glioma	25	9.38 × 10^−28^
hsa05215	Prostate cancer	27	3.62 × 10^−26^
hsa04380	Osteoclast differentiation	30	6.93 × 10^−26^

**Table 6:** The top 12 GO biological processes in sub-iliac lymph nodes collected from fed beef cattle and cull dairy cattle. The statistically significant peptides were generated from the comparison of feedlot and cull dairy cattle sub-iliac lymph nodes using the online STRING database to generate a list of representative GO terms. The top 12 terms are listed. The pathways in bold are those involved in the immune response (total immune peptides: 430).

GO ID	Term	# Peptides	*p*-Value (FDR)
**GO:0045087**	**innate immune response**	**103**	**7.52 × 10^−58^**
GO:0007169	transmembrane receptor protein tyrosine kinase signaling pathway	83	1.25 × 10^−54^
GO:0007167	enzyme linked receptor protein signaling pathway	91	9.42 × 10^−53^
**GO:0050776**	**regulation of immune response**	**88**	**5.12 × 10^−48^**
**GO:0006955**	**immune response**	**108**	**5.65 × 10^−48^**
**GO:0002764**	**immune response-regulating signaling pathway**	**73**	**5.65 × 10^−48^**
GO:0051246	regulation of protein metabolic process	124	2.86 × 10^−46^
GO:0032268	regulation of cellular protein metabolic process	111	7.15 × 10^−45^
GO:1901700	response to oxygen-containing compound	98	3.30 × 10^−44^
GO:0071310	cellular response to organic substance	113	3.30 × 10^−44^
**GO:0038093**	**Fc receptor signaling pathway**	**58**	**7.17 × 10^−44^**
GO:1901699	cellular response to nitrogen compound	67	2.13 × 10^−43^

**Table 7:** Details of summer cattle that had either high or low concentrations of *Salmonella* in the sub-iliac lymph nodes, and winter cattle, that were positive or negative for *Salmonella* in the sub-iliac lymph node collected for kinome analysis.

				*Salmonella*	
Sample ID	Date	Source	Season	Concentration (CFU (log_10_)/g Lymph Node)	Prevalence
8XC	28 July 2014	Feedlot	summer	1.5	pos
9XC	28 July 2014	Feedlot	summer	2.3	pos
3XC	28 July 2014	Feedlot	summer	0.1	pos
5XC	28 July 2014	Feedlot	summer	0.2	pos
10XC	28 July 2014	Feedlot	summer	2.8	pos
5	19 January 2015	Feedlot	winter	neg	neg
2	19 January 2015	Feedlot	winter	0.8	pos
1	19 January 2015	Feedlot	winter	0.1	pos
27	19 January 2015	Feedlot	winter	0.1	pos
30	19 January 2015	Feedlot	winter	neg	neg

**Table 8:** Top 12 KEGG pathways in the sub-iliac lymph nodes collected from feedlot cattle at slaughter in the summer and winter months. A list of representive pathways was generated by utilizing the statistically significant peptides from the comparison and inputting these peptides into the online STRING database. Presented below are the top 12 pathways. The bold pathways are those involved in the immune response (total immune peptides: 146).

KEGG ID	Pathway Name	# Peptides	*p*-Value (FDR)
hsa04722	Neurotrophin signaling pathway	43	2.74 × 10^−42^
hsa04010	MAPK signaling pathway	54	4.76 × 10^−40^
hsa04910	Insulin signaling pathway	42	4.76 × 10^−40^
hsa05200	Pathways in cancer	57	5.85 × 10^−39^
hsa04012	ErbB signaling pathway	34	1.73 × 10^−36^
**hsa04510**	**Focal adhesion**	**45**	**9.91 × 10^−36^**
**hsa04660**	**T cell receptor signaling pathway**	**34**	**1.72 × 10^−32^**
hsa05214	Glioma	26	1.46 × 10^−28^
**hsa04062**	**Chemokine signaling pathway**	**38**	**1.46 × 10^−28^**
hsa05215	Prostate cancer	28	9.67 × 10^−27^
**hsa04620**	**Toll-like receptor signaling pathway**	**29**	**2.12 × 10^−26^**
hsa05220	Chronic myeloid leukemia	25	1.97 × 10^−25^

**Table 9:** Top 12 GO biological processes in the sub-iliac lymph nodes collected from feedlot cattle in the summer and winter months. A list of representative GO terms was generated by taking the statistically significant peptides from the comparison and inputting this peptide list into the online STRING database. Presented below are the top 12 terms from the input data. The pathways in bold are those involved in the immune response (total immune peptides 406).

GO ID	Term	# Peptides	*p*-Value (FDR)
**GO:0045087**	**innate immune response**	**111**	**6.70 × 10^−63^**
GO:0007169	transmembrane receptor protein tyrosine kinase signaling pathway	88	1.05 × 10^−57^
**GO:0050776**	**regulation of immune response**	**99**	**1.89 × 10^−56^**
GO:0007167	enzyme linked receptor protein signaling pathway	97	2.61 × 10^−56^
**GO:0002764**	**immune response-regulating signaling pathway**	**80**	**1.26 × 10^−53^**
**GO:0006955**	**immune response**	**116**	**8.50 × 10^−52^**
GO:0071310	cellular response to organic substance	125	1.16 × 10^−50^
GO:0009967	positive regulation of signal transduction	100	5.06 × 10^−47^
GO:0048584	positive regulation of response to stimulus	118	5.16 × 10^−47^
GO:0051246	regulation of protein metabolic process	129	1.26 × 10^−46^
GO:0010033	response to organic substance	137	1.36 × 10^−45^
GO:0070887	cellular response to chemical stimulus	130	6.23 × 10^−45^

**Table 10:** Details of peripheral lymph nodes from cattle that were experimentally or naturally infected with *Salmonella* collected for kinome analysis.

				*Salmonella*	
Sample ID	Date	Source/ Cattle Type	Season	Concentration (CFU (log_10_)/g Lymph Node)	Prevalence
20	19 January 2015	Feedlot/ Steers	winter	0.1	pos
2	19 January 2015	Feedlot/ Steers	winter	0.8	pos
1	19 January 2015	Feedlot/ Steers	winter	0.1	pos
13	19 January 2015	Feedlot/ Steers	winter	0.6	pos
27	19 January 2015	Feedlot/ Steers	winter	0.1	pos
10	19 January 2015	Feedlot/ Steers	winter	0.1	pos
1 R	20 January 2015	USDA/ Holstein steers	winter	1.5	pos
1 L	20 January 2015	USDA/ Holstein steers	winter	1.2	pos
5 R	20 January 2015	USDA/ Holstein steers	winter	1.1	pos
5 L	20 January 2015	USDA/ Holstein steers	winter	1	pos
14 R	20 January 2015	USDA/ Holstein steers	winter	1.1	pos
14 L	20 January 2015	USDA/ Holstein steers	winter	1.1	pos

**Table 11:** The top 12 KEGG pathways in sub-iliac lymph nodes collected from experimentally and naturally infected cattle. A list of representative pathways is generated using the statistically significant peptides from the comparison and inputting this peptide list into the online STRING database. Presented below are the top 12 pathways. Pathways in bold are those involved in the immune response (total immune peptides: 161).

KEGG ID	Pathway Name	# Peptides	*p*-Value (FDR)
hsa04910	Insulin signaling pathway	52	1.14 × 10^−52^
hsa05200	Pathways in cancer	67	1.71 × 10^−47^
hsa04012	ErbB signaling pathway	40	1.61 × 10^−44^
hsa04722	Neurotrophin signaling pathway	45	8.03 × 10^−44^
hsa04010	MAPK signaling pathway	58	1.66 × 10^−42^
**hsa04510**	**Focal adhesion**	**50**	**6.56 × 10^−40^**
**hsa04660**	**T cell receptor signaling pathway**	**38**	**1.02 × 10^−36^**
hsa05214	Glioma	28	1.53 × 10^−30^
hsa05215	Prostate cancer	31	1.02 × 10^−29^
**hsa04062**	**Chemokine signaling pathway**	**40**	**3.23 × 10^−29^**
**hsa05160**	**Hepatitis C**	**33**	**2.15 × 10^−26^**
hsa04810	Regulation of actin cytoskeleton	39	4.89 × 10^−26^

**Table 12:** The top 12 GO biological processes in sub-iliac lymph nodes collected from cattle that were experimentally and naturally infected with *Salmonella.* A list of representative GO terms was generated by taking the statistically significant peptides from the comparison and inputting this peptide list into the online STRING database. Presented below are the top 12 terms from the input data. The pathways in bold are those involved in the immune response (total immune peptides: 652).

GO ID	Term	# Peptides	*p*-Value (FDR)
**GO:0045087**	**innate immune response**	**127**	**2.70 × 10^−74^**
**GO:0050776**	**regulation of immune response**	**114**	**3.10 × 10^−67^**
**GO:0002764**	**immune response-regulating signaling pathway**	**92**	**1.70 × 10^−63^**
GO:0007169	transmembrane receptor protein tyrosine kinase signaling pathway	95	1.22 × 10^−61^
GO:0007167	enzyme linked receptor protein signaling pathway	105	4.90 × 10^−60^
**GO:0006955**	**immune response**	**131**	**1.01 × 10^−59^**
GO:0071310	cellular response to organic substance	139	1.46 × 10^−56^
GO:1901700	response to oxygen-containing compound	120	5.69 × 10^−56^
**GO:0002682**	**regulation of immune system process**	**119**	**1.12 × 10^−54^**
**GO:0038093**	**Fc receptor signaling pathway**	**69**	**8.27 × 10^−53^**
GO:0042325	regulation of phosphorylation	114	1.49 × 10^−52^
GO:0051246	regulation of protein metabolic process	144	1.49 × 10^−52^

## Data Availability

Not Applicable.

## Supplementary Materials

The following supporting information can be downloaded at: https://www.mdpi.com/article/10.3390/microorganisms10010120/s1, Figure S1. A heatmap and cluster analysis representing all peripheral lymph nodes samples including cattle type (cull dairy or feedlot), season (summer and winter), infection status (*Salmonella* positive or negative) and source of *Salmonella* (experimentally or naturally infected). Each column represents a tissue sample; each row is a peptide. Red represents relative increase in phosphorylation and green relative decrease. Table S1. Statistically significant differentially phosphorylated peptides for each cattle experimental group.
