# Supplementary material for: Kinome Analysis of Cattle Peripheral Lymph Nodes to Elucidate Differential Response to Salmonella spp
Source: Microorganisms. 2022 Jan 7;10(1):120. doi: 10.3390/microorganisms10010120 (PMC8779847; doi:10.3390/microorganisms10010120)
Supplement: Supplementary file 1 [file microorganisms-10-00120-s001.zip › Supplementary Figure S1.pdf]

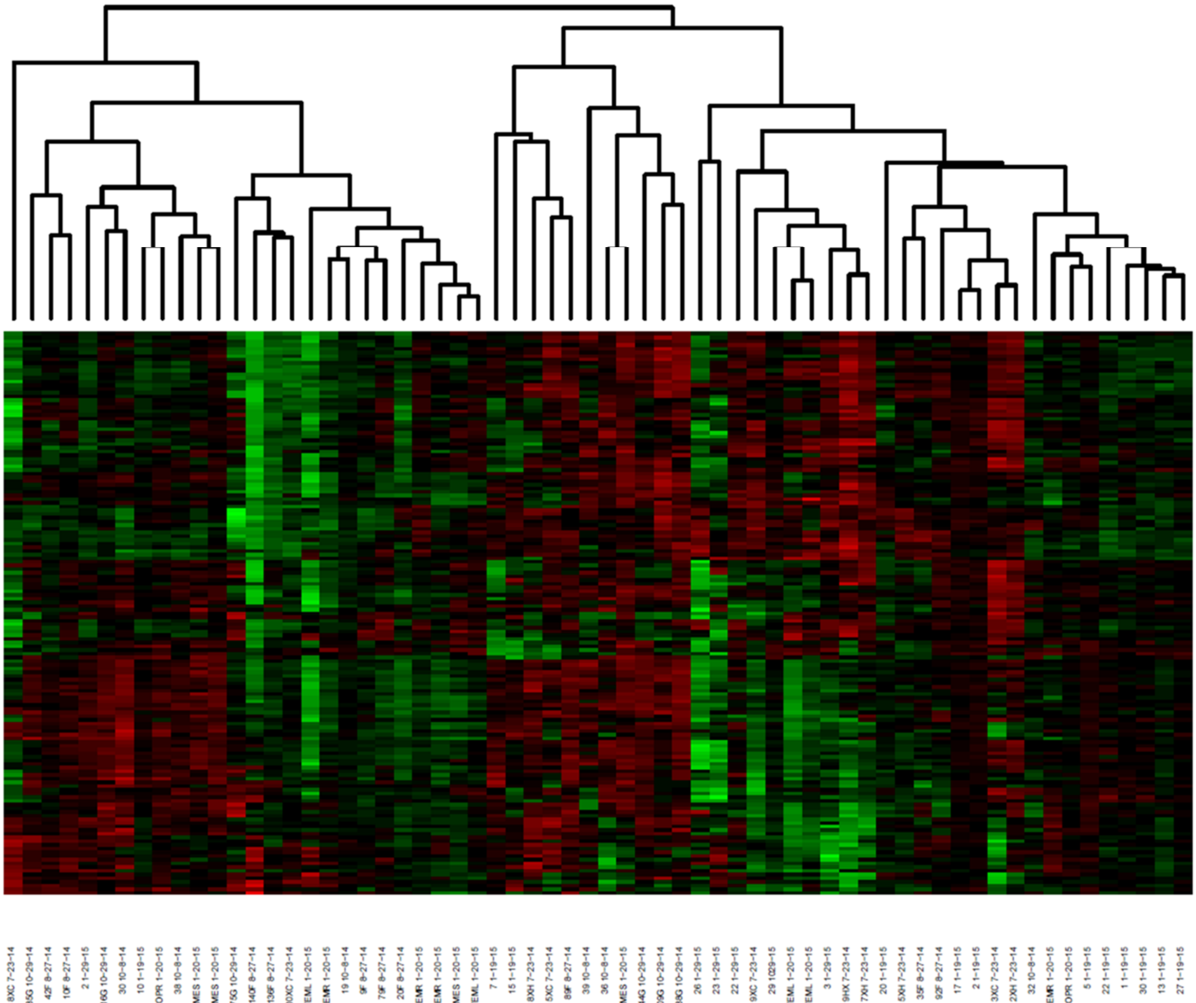

Figure S1. A heatmap and cluster analysis representing all peripheral lymph nodes samples including cattle type (cull dairy or feedlot), season (summer and winter), infection status (*Salmonella* positive or negative) and source of *Salmonella* (experimentally- or naturally-infected). Each column represents a tissue sample, each row is a peptide. Red represents relative increase in phosphorylation and green relative decrease.
